# A qualitative study on the perspectives of Turkish mothers and grandmothers in the Netherlands regarding the influence of grandmothers on health related practices in the first 1000 days of a child’s life

**DOI:** 10.1186/s12889-022-13768-8

**Published:** 2022-07-16

**Authors:** Gülcan Bektas, Femke Boelsma, Meryem Gündüz, Eva N. Klaassen, Jacob C. Seidell, Carline L. Wesdorp, S. Coosje Dijkstra

**Affiliations:** 1 Department of Health Sciences, Faculty of Science, Vrije Universiteit Amsterdam, Amsterdam Public Health Research Institute, Van der Boechorstraat 7, BT 1081 Amsterdam, the Netherlands; 2 Faculty of Sports and Nutrition, Centre of Expertise Urban Vitality, Amsterdam University of Applied Sciences, Dokter Meurerlaan 8, 1067 SM Amsterdam, the Netherlands; 3 Department of Human Movement Sciences, Faculty of Behavioural and Movement Sciences, Vrije Universiteit Amsterdam, Van der Boechorstraat 7, 1081 BT Amsterdam, the Netherlands

**Keywords:** Early childhood, Health promotion, Grandmothers, Parenting, Socio-cultural beliefs

## Abstract

**Background:**

Given the importance of the first 1000 days of a child’s life in terms of laying the foundations for healthy growth and development, parents are a logical target group for supporting health-related practices with regard to young children. However, little attention is paid to the influence of the wider social community on the health and development of young children during this crucial period. This includes grandmothers, who often have a significant influence on health-related practices of their grandchildren. The aim of this study was therefore to explore the influence of grandmothers on health related practices of their grandchildren during the first 1000 days, from the perspectives of both grandmothers and mothers with a Turkish background.

**Method:**

This qualitative study in the Netherlands collected data during focus group discussions with grandmothers (*N =* 3), interviews with grandmothers (*N =* 18) and interviews with mothers (*N =* 16), all with a Turkish background. Data was collected in the period between June 2019 and April 2021 and analysed using a thematic content analysis.

**Results:**

The influence of grandmothers and the wider social community on health related practices during the first 1000 days of a child’s life is substantial and self-evident. The support of grandmothers is often rooted in various socio-cultural norms and practices. The mothers of young children can experience the guidance and pressure they receive from grandmothers and the wider social community as quite stressful. Conflicting views and practices tend to arise between grandmothers and mothers when a grandmother babysits. Both mothers and grandmothers often find it difficult to discuss these differences openly, for fear this might lead to a family conflict.

**Conclusion:**

This study shows that grandmothers and the wider social community play an influential role in supporting a healthy first 1000 days of a child’s life. The strong involvement of grandmothers may lead to tension between the mothers and grandmothers when their ideas about healthy practices are not in agreement and may lead to unhealthy practices. In targeting this wider social community, it is important to consider the various socio-cultural factors that underlie the advice, support, practices and beliefs of the individuals involved.

**Supplementary Information:**

The online version contains supplementary material available at 10.1186/s12889-022-13768-8.

## Introduction

The foundation for the healthy growth and development of a child is laid during the first 1000 days of its life – the period from conception until the child’s second birthday [1–5]. This period is an important time for establishing health-related behaviours such as dietary habits, physical activity and sleep patterns [6–8]. Parental practices during the first 1000 days are important in terms of influencing a child’s health as they serve as an example and determine the health-related behaviours of their children [9–11]. In light of these considerations, parents are usually the main target group of initiatives aimed at supporting healthy behaviours in young children.

However, less attention seems to be paid to the influence of the parents’ wider social circle on healthy practices during the first 1000 days, such as family or friends and the wider community, notwithstanding the important role these people play in providing parents with practical support and information that indirectly influences their health-related practices [12–14]. An important source of caregiving in this wider social environment can be the grandparents, since the traditional role of the mother as primary caregiver has changed due to the relatively short maternity leave granted to working mothers [15, 16]; grandmothers especially are fulfilling the role of alternative caregiver for their grandchildren [17–19]. In many countries, cultural and traditional structures give grandparents an important position as caregivers, since a child’s upbringing is considered a collective responsibility, which means that alloparents (e.g. grandparents, aunts, uncles) can influence the child’s development, health, and well-being [20, 21].

The impact of grandparents on healthy practices with regard to young children has been the subject of various studies which show for example that grandparents are more likely to encourage unhealthy practices in the first 1000 days, such as giving unhealthy food as a treat and allowing the child more screen time than recommended [22–27]. A British study found that children who were mainly cared for by their grandmothers between the ages of 9 months and 3 years were more likely to be overweight at the age of three than children who were cared for by their parents [28]. However, a study from Sweden indicated a different association between the involvement and support of grandparents and childhood obesity, indicating that higher levels of grandparental support could be preventive against childhood obesity [29]. These different results emphasise the complexity of the influence that grandparents can have on the health of young children.

In the Netherlands, people with a Turkish background form the largest group with a non-Dutch background [30]. Research into the influence of grandmothers with a Turkish background on the health of children during the first 1000 days has been limited, though one study has found that the risk of being overweight at the age of 2 is between two and four times higher for a child from a Turkish background than a child from a Dutch background [31]. In order to gain a better understanding of the practices of grandparents, it may be helpful to look at the socio-cultural norms, traditions and preferences that may play an underlying role [12, 19, 32]. For instance, a Turkish study reported that it is a common traditional practice for grandmothers and relatives to provide food the mother craves during pregnancy, because they believe this will help prevent certain deficiencies in the baby [33]. As childrearing practices, beliefs and traditions are determined by the structure of the family systems which are embedded in cultural systems and shaped by the physical and social settings in which they live [34], it is important to consider the socio-cultural dynamics that underlie the health-related practices of both parents and grandparents, and the interactions at play during the child’s first 1000 days.

In the traditional Turkish family, respect for authority is strongly valued, which means that elderly such as grandparents should be respected for their age, knowledge and experience. This reinforces parents’ dependency and obedience to grandparents [35, 36]. Although the norms, values and parental practices of second generation Turkish migrant parents are more acquainted with the Dutch society, they still posit a high value on dependency and obedience to parents because of being raised up by parents who retain the family values, traditions and culture of their origin country [37, 38]. As a result of these socio-cultural norms and family dynamics, parents can be hesitant to explain to grandparents that there are certain health-related practices they do not agree with [24, 25, 33] and may feel pressured to go along with the preferences of their social community and culture [22]. Further, within the Turkish family system it is often acknowledged that women are more likely to provide care than men, and are therefore more involved and responsible to take care for the child, which makes them more influential [35, 37]. It is therefore important to have a better understanding of the influence of grandmothers with a Turkish background, since they play a key role in their grandchildren’s upbringing and the health-related practices that affect their future [13, 22, 39].

In this study, we will focus on exploring the influence of grandmothers with a Turkish background on the health-related practices in the first 1000 days of a child’s life, seen from the perspectives of both mothers and grandmothers. A better understanding of the socio-cultural factors at play can contribute to optimising ways to promote a healthy first 1000 days for children.

## Methods

### Study design

This study is part of the Food4Smiles research project, which aims to contribute to the healthy growth and development of children during the first 1000 days of life, focusing on a relatively low-income, multi-ethnic neighbourhood in Amsterdam, the Netherlands. As in all cities, there are health differences between the various neighbourhoods in Amsterdam, including differences in the prevalence of childhood obesity. For example, 1 in 4 children is overweight in the neighbourhood under study compared to 1 in 10 in other neighbourhoods of Amsterdam [40], and 29.8% of these children have a Turkish background [41].

The current study is based on the findings from our previous explorative study [22], which indicated that the wider social community, and grandmothers in particular, have an influence on health-related practices affecting young children. In this study, we explore *how* grandmothers influence health-related practices in the first 1000 days of their grandchild’s life. Due to the exploratory nature of the study, we selected a qualitative study design that involved the use of focus groups and semi-structured interviews with mothers and grandmothers with a Turkish background, as we observed in our previous study in this area of research that grandmothers are pivotal contributors in childcare [22]. The combination of interviews and focus groups allowed us to achieve richer information.

We began by conducting three focus groups with grandmothers (*N =* 11) before the outbreak of the COVID-19 pandemic. Subsequently, due to the COVID-19 restrictions on group size we were not able to conduct focus groups with mothers and continued by holding interviews with grandmothers (*N =* 18) and interviews with mothers (*N =* 16).

### Recruitment

Recruitment took place between June 2019 and April 2021. Turkish mothers and grandmothers with a (grand) child aged 0–4 years, who lived in the Netherlands were recruited to take part in this study. To make sure that grandmothers were actively involved in raising the grandchildren the inclusion criterion was that grandmothers have to look after their grandchild for at least 2 times a week. We also included only mothers whose parents were looking after their child for at least 2 times a week. The recruitment was done through a variety of channels, including posting the study on existing parents’ app groups or other social media channels relating to the project (e.g. Instagram), and personally approaching potential participants in neighbourhood playgrounds or parks. Additionally, two researchers (GB and MG) lived in the neighbourhood under study and had therefore a personal network to address for the recruitment. Subsequently, we used snowball sampling: asking the respondents we had recruited whether they knew of other potential respondents. Potential respondents were approached and invited by the mothers and grandmothers to take part in the study, and after their agreement contact details were shared with the researchers in order to make an appointment for an interview. The mothers and grandmothers included as respondents in the study were all able to speak Dutch or Turkish during their interview and focus groups discussions, as these were the languages spoken by the principal researcher (GB) and two research assistants (MG & EK).

### Data collection

First, in June 2019 focus group sessions (*N =* 3) were held with grandmothers. These sessions were moderated by the principal researcher (GB) and were designed to explore the grandmothers’ perceptions and experiences regarding the childcare they provide during the first 1000 days of their grandchild’s life, but also to obtain a greater insight into the relevant socio-cultural dynamics. During the focus group discussions, a topic list was used as a guideline to facilitate the group’s conversations. Relevant topics raised in the literature were included in the topic list: the role played by grandmothers; grandparental perspectives on health-related behaviours with regard to young children (i.e. feeding, sleeping, physical activity and screen time); Turkish perspectives and traditions regarding pregnancy, the health status of a baby/infant; child-rearing practices; health across different cultures; and the communication between grandmothers and their daughter/son (in-law) regarding practices that affect child health. See Additional file 1: Appendix A for an overview of the topics discussed during the focus group discussions with grandmothers. The focus groups lasted around 60 minutes each and were all conducted in Turkish. The conversation was transcribed verbatim and later translated into Dutch by the principal researcher (GB).

Second, the semi-structured interviews with 18 grandmothers and 16 mothers were conducted between November 2020 and April 2021. Eight of them were pairs (mothers and grandmothers from the same family) and eighteen were individual mothers (*n =* 8) and grandmothers (*n =* 10), who had no direct family relation. For the interviews with the grandmothers, we used the same topic list that was used in the focus group discussions. We adapted this topic list for the mothers by framing the questions from their perspective, in order to make sure that both the grandmothers and the mothers were invited to reflect on the same topics (see Additional file 2: Appendix B). The interviews lasted between 40 and 100 minutes. Due to the COVID-19 restrictions, the respondents were given the option of conducting the interviews face-to-face (*N =* 19), via video call (i.e. Zoom or WhatsApp) (*N =* 17) or via telephone (*N =* 1). The interviews with the mothers and grandmothers were conducted in either Dutch (*N =* 17) or Turkish (*N =* 17). The Turkish interviews were also transcribed verbatim and translated into Dutch by the research assistant (MG) and checked by the principal researcher (GB), who are both bilingual in Dutch and Turkish, in order to increase validity.

All of the focus groups and interviews were recorded after obtaining the participants’ consent. Every interview and focus group discussion ended with additional questions about the age of the respondents, the highest level of education they had attained, employment status, number and age of (grand) children, and years of residence in the Netherlands. Educational level was classified as no education/primary education (low); lower secondary education/higher secondary education (middle); higher vocational college/university (high). All of the grandmothers were born in Turkey and the mean of their years of residence in the Netherlands was 37.8 with a range of 4-51 years. All of the mothers were born and raised in the Netherlands, with exception of one mother who had been living in the Netherlands for 14 years. Table 1 presents the characteristics of the respondents.

**Table 1:** Characteristics of the respondents who participated in the study (total *N =* 45)

	Mothers (***N =*** 16)	Grandmothers (***N =*** 29)
**Age in years, mean (range)**	31.2 (25-40)	55.9 (42-77)
**Education level, n (%)**		
Low	2 (12.4)	17 (58.6)
Middle	7 (43.8)	11 (37.9)
High	7 (43.8)	0 (0)
Unknown	0 (0)	1 (3.5)
**Employment status, n (%)**		
Employed	10 (22.2)	6 (20.7)
Unemployed	6 (77.8)	23 (79.3)
**Living with, n (%)**		
Extended family	5 (31.3)	3 (10.3)
Nuclear family	11 (68.7)	26 (89.7)
**Age of infant in months, mean (range)**	21.9 (3-36)	22.1 (0-48)
**Age categories of infants, n (%)**		
0-6 months	1 (5.6)	4 (8.2)
6-12 months	4 (22.2)	11 (22.5)
12-24 months	4 (22.2)	13 (26.5)
24-36 months	9 (50.0)	13 (26.5)
36-48 months	0 (0)	8 (16.3)

### Data analysis

A thematic content analysis was done with the aim of coding the experiences and perspectives of the respondents as openly as possible. The computer programme ATLAS.ti [42] was used for this process. In accordance with the principles of inductive thematic analysis, the transcripts were coded and checked independently by two researchers (GB and FB) to increase reliability and ensure uniform coding [43]. They both identified inductive recurring categories in the interviews in order to develop conceptualizations of the tentative relations within and between categories. The answers and themes that emerged from the interviews of the pairs and the interviews of the individual grandmothers and mothers were compared. We did not observe important differences and therefore we analysed all the respondents as one group by focusing on the perspectives of mothers and grandmothers rather than the relational dynamics within the family structures. Subsequently, the common categories were discussed by both researchers to detect consistent and overarching themes. While consensus was reached on most topics, some topics were explored further before consensus was found. Through a process of consultation, we subsequently established a consensus on the relevant categories and key findings. See Additional file 3: Appendix C for an overview of the development of the themes.

### Consent and ethical considerations

All of the respondents who took part in the study received a letter informing them about the study and provided a written statement of informed consent. Since some of the respondents were not able to understand Dutch, the information letter and informed consent were translated into Turkish to ensure that they were able to fully understand the aim of the study and give consent for data collection. The translation was carried out by two Turkish-speaking researchers (GB & MG) to increase the credibility of the translation. Additionally, since one of the recruitment strategies was addressing the personal network of the researchers, respondents were interviewed by a researcher who was unknown for them in order to ensure privacy. The interviews were transcribed verbatim and anonymised by removing identifying characteristics from the data, and the data was analysed afterwards. The study was approved by the Medical Ethical Committee of Amsterdam UMC (VUmc location).

## Results

Four themes were derived from the analysis: (1) ‘The support and cogent advice of grandmothers and the wider social community during the first 1000 days is self-evident and often rooted in socio-cultural beliefs and practices’; (2) ‘Grandmothers and the wider social community actively encourage mothers to breastfeed’; (3) ‘Grandmothers often deviate from their grandchildren’s daily routine when they are babysitting’; and (4) ‘The communication between mothers and grandmothers about differences of opinion regarding health-related practices is perceived as difficult.’

### The support and cogent advice of grandmothers and the wider social community during the first 1000 days is self-evident and often rooted in socio-cultural beliefs and practices

The mothers described a situation in which many people in their wider social community, particularly grandmothers, were very involved during the first 1000 days of their child’s life. The wider social community referred to the involvement and influence of both kin (e.g. aunts, uncles, cousins) and non-kin people (e.g. friends, neighbours or members of the community). This community seemed to feel they had the right to have a say in how the baby is raised and looked after, and advice was readily given. Two mothers described this as:

*“In our [social community, ed.] everybody has the right to voice an opinion about the care of the baby. [ …*] *Everybody interferes.” (I-M6).*

*“In our [social community, ed.] you don’t need to ask for advice: advice is given.” (I-M11).*

The grandmothers explained that they were often very much involved in the well-being of their grandchildren, and said they offered well-meaning advice and tips. They explained that they provide support during the first 40 days postpartum in particular, a period widely regarded in Turkish culture as a time when mothers should receive extra help and check-ups from the social community. The grandmothers said they would like mothers to trust their knowledge and experience, because they believe this would benefit the health and well-being of the child. One grandmother said:

*“Take your mother’s advice seriously, because it contains invaluable things that you cannot get from a gynaecologist, a midwife or a child health centre and it’s all for the good of your child. So listen to us, because we too know a little bit about these things.” (I-GM-15).*

Mothers described grandparents, especially grandmothers, as playing a vital role in supporting their family, helping to host visits by family members and cook meals during the postpartum period, and generally being a source of useful information. One mother said:

*“The role of grandparents … I think the role of grandparents with regard to our children and family … I couldn’t do without them. We couldn’t do without them. My mother-in-law often sends us meals … not all the time but often. Very often, and that’s so nice.” (I-M15).*

While the mothers often appreciated the advice they were given, some mothers said that dealing with a grandmother’s advice when they did not agree with it could be a drain on their energy, because some grandmothers were very persistent. One mother described how exhausting it could be when her own mother and mother-in-law kept insisting that things should be done their way:

*“Yes, but they kept going on and on about it, and that made it difficult because they kept repeating it. [ …] I kept saying, ‘OK, OK’ but they just kept on going. [ …] You just don’t have the energy to keep telling them the same thing. You’ve just had a baby, so you’re tired, you are still recovering.” (I-M12).*

The mothers and grandmothers described various traditional socio-cultural practices and beliefs that play a role in the advice and support that parents receive from their social community during the first 1000 days. According to grandmothers and mothers, it is common in Turkish culture to prefer a chubbier baby. A chubby baby is considered to be well-fed and well-cared for. One mother said about this:

*“Yes, in our culture it’s a case of: the bigger the baby, the better. Being fat is a sign of prosperity. That’s something from back in the old days.” (I-M9).*

Grandmothers explained that a chubby, well-fed child shows their community that they love their children and look after them well. One grandmother explained:

*“In the eyes of us Turks, if a child is too thin that’s because it’s not well-fed or well cared for. I think that’s why we think a chubby baby is a baby that’s well looked after. So we say: ‘Oh, that mother takes good care of her baby, she is well fed.’ That’s how we see it. I know it isn’t really true, but everyone thinks a chubby baby is much cuter.” (I-GM11).*

Grandmothers in particular said they thought a child should be chubby and believed their grandchildren should not be too thin, associating low body weight with the danger of falling ill. One grandmother said:

*“It’s about making sure the children eat well, so that if they get sick, they won’t immediately lose weight. My daughter doesn’t want them to eat too much, but I want them to eat a bit more.” (I-GM18).*

However, some grandmothers said a chubby child is not necessarily a healthy child and believed that the most important thing is that a child gets enough to eat. Grandmothers found that by seeking out more up-to-date knowledge about health-related practices, they had become more aware that being overweight is not healthy. Even though some grandmothers said they had changed their views about what is a healthy weight for a child, they all found their own grandchild too thin and wanted them to put on a little weight. Another social-cultural issue about raising children that came to the fore was the acceptability of leaving a child to cry. The mothers, and especially the grandmothers, seemed to believe that if a young child is crying, it must be hungry and/or you should give it what it wants. One mother said in this regard:

*“But they [the grandparents, ed.] want to raise the child the Turkish way, and give them everything they want so they don’t cry.” (I-M6).*

Many grandmothers remarked that they did not want their grandchild to cry. Some grandmothers gave this as a reason for wanting to please their grandchildren and give them everything they wanted – in most cases something nice to eat –to stop them crying. On this topic, one grandmother said:

*“For example, when we are in a shop or a grocery store we buy everything the children want to stop them crying. We buy it so that the child won’t cry, otherwise we feel like everyone is staring at us.” (I-GM8).*

Lastly, a social-cultural idea with regard to raising a child seems to be that grandparents are allowed to indulge and spoil their grandchildren. Most grandmothers said they wanted their grandchildren to have fun and enjoy their time with their grandparents to make them feel loved and to make the future bond between them and their grandchildren stronger. This was mostly done by indulging or rewarding the children. Grandmothers said they mostly rewarded their grandchildren with food they like (e.g. pizza or crisps), toys and/or extra screen time.

*“When they’re at grandma’s, things can be different or less strict. It should be fun, and it’s up to the parents to apply their own rules at home. He only comes to my house twice a week, so I can do whatever I want. And I think I have the right to spoil him.” (FG-GM1).*

According to the mothers, grandfathers were especially inclined to give their grandchildren sweets, and the mothers found it difficult to discuss this with them. One mother described how her father-in-law continued to give the children sweets, despite being asked not to:

*“He [father-in-law, ed.] kept challenging me. And if I looked at him as if to say, ‘No, I don’t want that. He needs to eat first. He’s a one-year-old baby, he doesn’t need a chocolate bar or any of those sweets. He doesn’t need them.’ [ …] When they come to visit they all bring sweets, and when we go to see them there are always sweets around. And when I see my father-in-law giving him sweets, I say: ‘No papa, later’. And he says ‘Just one?’ And it usually ends up being two or three.”(I-M15).*

### Grandmothers and the wider social community actively encourage mothers to breastfeed

Mothers explained that grandmothers and other people in their social community were actively encouraging them to breastfeed their baby as much and/or as long as possible until the age of 2. All of the mothers indicated that they wanted to breastfeed their baby. However, some mothers who had problems breastfeeding found themselves being pushed by others to not give up, which made them feel pressured. One mother said in this regard:

*“With my first child I felt like: if I don’t breastfeed it means that I’m an inferior mother. Everybody around me was saying: ‘Yes, you really need to breastfeed’. [ …] You just feel that pressure, it’s like you have to breastfeed.” (I-M7).*

Grandmothers expressed the view that mothers nowadays tend to stop breastfeeding very quickly and said they encouraged mothers to breastfeed as long as possible, preferably up to 2 years of age. Many of the grandmothers said breastfeeding has a religious significance: the principles of Islam state that every new-born infant has the right to be breastfed for up to 2 years if possible, and they also believed that children who were breastfed were more robust and healthy. As one grandmother put it:

*“I said to her, ‘Breastfeed and your child will be strong.’ And she said ‘I can’t because of my job and I’m not able to express milk.’ That’s why her child is so thin.” (I-GM5).*

To encourage breastfeeding, many grandmothers said they prepared or bought lots of sweet foods and drinks such as Turkish delight, dried figs and dates, fruit syrup or compote. Based on their own experience, they believed that consuming a lot of sweet drinks and food increases milk production. One grandmother said in this regard:

*“I gave that advice, because milk production increases when you consume sweet things. They say things like fruit syrup or compote are also good. And they also tell you to eat a lot of fruit so that your child will be sweet and beautiful.” (I-GM14).*

Mothers and grandmothers also noted that grandmothers and others in the social community were very concerned about whether the baby was satiated by the quality and quantity of the breastfeeding. Mothers were often questioned by others about whether their breastmilk was enough for the child, and were sometimes pushed to supplement their breastfeeding with additional bottle-feeds to make sure the child did not go hungry. One grandmother said:

*“If the child is crying, he is probably still hungry. Just give him an extra bottle, it can’t hurt.” (IGM9).*

Mothers who decided to stop breastfeeding sometimes faced questions from other people in their social community about their reasons for stopping. This sometimes prompted feelings of failure or shame. One mother explained this as follows:

*“That was a difficult thing in our culture. To keep on hearing, ‘Yes, but why did you stop breastfeeding so early?’ It’s like you’re being handed a burden of shame. Not that I was ashamed of it, but it’s as if that’s being imposed on you: ‘How come you only breastfed for six weeks? You should be ashamed of yourself. Why is she being bottle-fed?’ [ …] My uncle’s wife also made a remark along the lines of ‘Hey, why aren’t you breastfeeding anymore?’” (I-M4).*

### Grandmothers often deviate from their grandchildren’s daily routine when they are babysitting

According to some mothers, the grandmothers were happy to adhere to the daily routine and health-related practices of the mother when they were babysitting, while in the experience of others, the grandmothers did not stick to the mother’s daily routines and health-related practices. One mother said in this regard:

*“In the beginning I typed and printed out a schedule which I gave [her, ed.]: ‘This is the schedule, please keep to it as much as possible.’ So I think that played a part, so she knows: ‘OK, she really wants her child to have a specific routine. I’d better not deviate from it too much.’ She does, of course, but not too much.” (I-M6).*

Almost all grandmothers said they followed their own routine, rules, health-related practices and experiences when babysitting their grandchild. Only a few grandmothers indicated that they follow the routines, rules and health-related practices of the child’s parents to the letter. Some grandmothers explained this by saying that mothers of the younger generation were much more modern than they were and that their child rearing was closer to the Dutch culture and norms. Most grandmothers said they therefore disagreed at times with the instructions or parenting practices of the child’s parents. In particular, the parents’ instructions about how and what to feed their child were seen as excessive and in many cases were ignored or led to a discussion with the parents. One grandmother said about this:

*“If I compare it with some children the same age in Turkey, for example, they eat everything. Those children are already eating cheese at 9 months. My grandchild doesn’t eat that yet. Why? ‘Because there’s salt in it.’ I understand, but it also makes me sad and sometimes I end up having words with my daughter about it.” (I-GM7).*

Despite instructions from parents, almost all grandmothers said that when they looked after their grandchild they gave the child food and drinks that the parents did not allow, such as processed foods (e.g. French fries, fried fish or sausage), sweets, chocolate, biscuits and/or crisps, fruit juice and hot chocolate. They believed that, as grandparents, it is perfectly acceptable to be less strict with a child than the parents. Grandmothers said they usually gave these foods secretly, without informing the parents. One grandmother said:

*“My son doesn’t want to give his son raw meat such as salami, sausage etc. He doesn’t do it himself and I know he doesn’t want me to do it, but I pretend I don’t know. [ …] When the parents aren’t around, I just give it to them and explain that it only happens when their parents are not around.” (FG2-GM1).*

Many mothers recognised that grandmothers tend to follow their own rules, especially with regard to food. Some mothers thought the grandmothers wanted to feed their child all day long. One mother said about this:

*“It really is non-stop food all day with grandpa and grandma [laughs]. It just doesn’t stop, you know [ …*]. *So every half hour they get a banana, a yoghurt or something or... It just never stops. It is eating continuously throughout the day. That’s pretty much how it goes.” (I-M10).*

Not all mothers found that the grandmothers pushed the children to eat. As one mother explains:

*“No, none of the parents on either side do that. Full is full, and if she doesn’t want to eat, then they stop. They don’t try to force her.” (I-M13).*

Some mothers prepared their child’s food for the day and gave this to the grandmothers when they were babysitting, in order to control the amount of food intake. One mother said:

*“I was quite strict the first year, especially when he was younger. Also about the kind of food he was allowed, supplementary foods and how many bottle-feeds he got. Because at the moment, he’s on the verge of being overweight. For the first year or 18 months, I think, I put everything he was allowed to eat that day in his bag, so I really controlled what he was allowed to eat and how much.” (I-M8).*

Regarding sleep, the sessions revealed some differences in opinion or practices between mothers and grandmothers. Most mothers said that the grandmothers stuck to the sleeping routine the mothers used for their child, but some explained that grandmothers were not all that concerned about putting the child to bed at a regular time. Mothers noticed that the grandmothers found it difficult to let the children cry for a while once they had been put to bed, because they were used to rocking the children to sleep on their legs. One mother said:

*“He always sleeps at the same time. And I said, ‘Don’t rock him, don’t rock him.’ She really had to unlearn that, get used to it. I said: ‘Let him cry for a bit, it’s OK. Just for ten minutes and if he keeps crying, then you can pick him up, you know.’” (I-R15).*

One grandmother said in this regard:

*“For example, I would like to rock her on my legs or rock her to sleep on my lap and I’m afraid to leave her alone, but her mother is the exact opposite. She tells me to put her to bed and close the door. I feel sorry for the child when it cries, but that’s how she wants it.” (I-GM2).*

### The communication between mothers and grandmothers about differences of opinion regarding health-related practices is perceived as complicated

As illustrated in the findings above, there are cases in which the mothers do not agree with the advice or health-related practices of the grandmothers and vice versa. Almost all of the grandmothers explained that they think the parents do not have the right to complain about their practices during babysitting and said they would feel disappointed if their daughter (in-law) were to correct them or complain about how they took care of the baby. Many grandmothers indicated that if the mother gave them instructions or tried to correct how they took care of their grandchild, they would stop babysitting and withdraw their support and help.

*“If she said anything about what I’m doing with the baby [ …*], *I’d be really disappointed. That would be a bit … ungrateful.” (FG3-GM2).*

Grandmothers also thought that the parents should look after the child themselves if they did not agree with their practices. As one grandmother put it:

*“She doesn’t have the right to say anything about it. And if she doesn’t like it then she should quit her job, stay at home and look after the baby herself.” (FG1-GM5).*

Many mothers found that it could sometimes be quite difficult to counter the advice or practices of the grandmothers. Most mothers indicated that they found it easier to discuss aspects of the child’s upbringing with their own mother, rather than their mother-in-law. As one mother explained:

*“To my own family I could maybe speak up more, to my direct family. But I am not going to tell my in-laws, ‘Thanks for the well-meaning advice, guys, but give it a rest will you …*? *’ you know?”(I-R4).*

One of the mothers explained that her own mother-in-law was very open to suggestions, but she also observed that this was not typical of other grandmothers:

*“I am very lucky with my mother-in law, she is someone who is open to new information. And often, in Turkish culture, people are not open to criticism, especially from a daughter-in-law, you know. People take it the wrong way and then it goes from being positive feedback to negative criticism. That’s how it comes across. And that’s what I’m trying to say: it’s not often you meet a mother-in-law like that, one to whom you can really say, ‘I’d kind of like things done this way because of such and such.’” (I-M15).*

Some mothers asked their husband to mediate if they had differences of opinion with their mother-in-law. One of the mothers illustrated this as follows:

*“With my mother-in-law it was a case of... We really had to say a couple of times, ‘Look, we don’t want to be questioned about breastfeeding,’ but she kept on asking. And so I asked my husband, ‘Can you please tell her sometimes too?’ And he did. So yes, I can’t say that she stopped right away, but there came a point when she understood that I really didn’t appreciate it.” (I-M1).*

Some grandmothers also remarked that fathers today are more engaged and involved in the upbringing and care of the child. According to grandmothers, in the past the mother had full responsibility for the children and other mothers were the only people they could talk to about raising young children, but now that fathers have become increasingly involved in the upbringing of their children, they are also included in discussions about their upbringing. One grandmother said in this regard:

*“I look at my own son and see that he bathes my grandchild, takes my grandchild to school, even though he works and his wife doesn’t. I think the new generation of fathers are very attentive and involved. And that means he also becomes someone to talk to about his children. In the past, fathers were invisible.” (FG2-GM1).*

A few mothers said they found it easier to resist unsolicited advice and social pressure from the social community and grandmothers when they felt more experienced as a mother themselves. They explained that once they had gained more parental experience, they felt that people in the wider social community were more inclined to let them do things their own way. One mother said:

*“With my second child I was a bit older, more mature. More experienced as well. You’re no longer a child who is afraid to hold a baby or feed it or something. You’re more mature, you’ve been through it once already. And you think: ‘No, I’m going to do what feels good for me and my child.’ And so you feel much more confident in saying: ‘No, she doesn’t want a drink … period!’” (I-M7).*

## Discussion

This qualitative study explored the influence of grandmothers on health-related practices with regard to their grandchildren during the first 1000 days of life, seen from the perspectives of both mothers and grandmothers with a Turkish background. We found that the influence of grandmothers, and the wider social community, during the first 1000 days is quite extensive and that this level of influence is seen as self-evident. Both mothers and grandmothers indicate that they had a lot in common during this period, but the extensive influence of grandmothers can also lead to unhealthy practices for the baby. A range of socio-cultural norms and practices play a role in the extensive influence of grandmothers, most notably cultural beliefs about the importance of breastfeeding, having a chubby baby and being respectful towards elders in the social community. We also found that there are sometimes conflicting views between mothers and grandmothers about practices relating to the baby’s health and that discussing these differing views is perceived as difficult, especially between mothers and their mothers-in-law.

The mothers in our study implied that grandmothers are very much involved in child rearing and consider looking after and protecting their grandchild to be partly their responsibility. Grandmothers confirmed this and saw themselves as pivotal contributors to the healthy growth and development of their grandchild. This active involvement derives from different socio-cultural factors, with great value being attached to one’s position within the community and the opinions of others. This can be explained by the fact that Turkish culture is known to be built around a collectivistic kinship structure that defines an individual’s role, responsibilities and obligations in relation to their affiliation with their family and community [21, 44]. This suggests that in collectivistic cultures there is a strong awareness of community values that demand high levels of group loyalty. Living up to the expectations of the community and abiding by its values and traditions are an important part of fitting in and being accepted [20, 45]. This leads families to raise their children as contributors to community life [45]. In our study, the watchful eye of the community is felt by both grandmothers and mothers, for example when they feel ashamed, uncomfortable or judged when a baby cries because their community could interpret this as a sign that they are not giving the baby the love and care it needs.

In a similar vein, we found that grandmothers were concerned about the health of the child and wanted to be sure that the child is well-fed, emphasising quantity rather than quality. It is well-known that grandparents encourage their grandchildren to eat larger portion sizes and give frequently food to them throughout the day. Studies described also that even though grandparents create a healthy food environment at their home by the availability and accessibility of it, they provide mostly discretionary foods to their grandchildren in order to indulge them [46, 47]. From an evolutionary perspective, a community’s concern with children being well-fed can be explained in terms of the need to ensure that young children survive and thrive in order to safeguard the future preservation and integrity of the community [48]. Traditionally, there are many environments in which food security can be compromised, and thin babies are more vulnerable to the effects of prolonged undernourishment or infections that cause weight loss. These considerations could provide a possible explanation for the preference for a well-fed and chubbier baby in certain cultures [22, 24, 49], especially when we take into consideration the fact that being overweight is seen as a sign of prosperity and good caregiving [50] and among grandmothers is associated with resilience to illness [22, 51]. We also observed that grandmothers attach great importance to breastfeeding. This has a foundation in the principles of Islam, which recommend that a new-born is breastfed for up to 2 years if possible [52]. This could be a reason why grandmothers push mothers to suckle their offspring frequently and advise them to eat specific foods that increase and stimulate breastmilk production, which could lead to higher levels of breastfeeding. It is described that many mothers appreciate and benefit from the support and instructions of the grandmothers regarding breastfeeding [53, 54]. However, their encouragement could also be experienced as pressure, which can lead to feeling insecure, confused and stressful [22, 55, 56], and could influence mothers’ mental health negatively [57, 58]. Additionally, grandmothers expressed concern as to whether a breastfed baby was satiated, which could result in pushing parents to overfeed their child by means of more frequent feeds, extra bottle-feeding and bigger serving sizes, as other studies have shown [59–61].

Both the mothers and grandmothers in our study explained that there were sometimes differences in the practices employed by mothers and grandmothers. For example, when babysitting, grandmothers tended to give food continuously throughout the day and let the children sleep whenever they wanted, which sometimes clashed with the food and sleep schedules set by the mothers. Generational differences in childcare practices have also been described in other studies, which found for example that grandparents like to spoil their grandchildren by giving them more control over what they eat, using food as a reward [26], using food to express their love for their grandchild [62], and having different views on sleeping practices than the parents [63]. These are not necessarily problematic practices, but given the extensive influence of grandmothers and the close interaction between grandmothers and the parents/grandchildren, they may lead to more structurally unhealthy practices with regard to young children and lead to friction between grandmothers and mothers when differences of opinion arise, as we have seen in our study.

In addition to the general differences in practices that can occur between generations, there also seem to be certain underlying Turkish beliefs and practices as outlined above, that influence the practices and experiences of both mothers and grandmothers. Other studies have also found that people with a Turkish background often maintain certain traditional parenting practices from their culture of origin [64–66]. Our study indicates that these socio-cultural beliefs and practices may be a stronger frame of reference for grandmothers than for mothers. The grandmothers in our study often came to the Netherlands in the 1960s and 1970s as part of a wave of immigrant workers and were almost all born in Turkey, which means they may hold stronger ties with the socio-cultural beliefs and practices of their country of origin than their daughters, who were usually born and raised in the Netherlands. These grandmothers seemed to believe that their daughters’ generation are more likely to follow Dutch child-rearing practices, which they themselves experience as more structured and rules-based. This is in line with another study in the Netherlands, which found that parenting practices (e.g. authoritative control) among second-generation Turkish migrants with young children are more in line with the practices of native Dutch parents [67]. Although, some child-rearing practices of grandparents are outdated or contradicts the recommendations given by child healthcare professionals, they are recognized by mothers as a source of knowledge, wisdom and experience in raising up and caring for child, and transferring culture and moral values [53, 68]. Especially younger and first-time mothers benefit most of grandmothers’ knowledge and experience, which has positive effects on their parental confidence as well as on their child’s health, and value their involvement and support during pregnancy and postpartum, and childrearing [69, 70].

Our study also shows that differences in practice between grandmothers and mothers are not always openly discussed between the two parties. Mothers often rely on the grandmothers for babysitting, and may therefore find it difficult to address certain practices for fear of negatively impacting the relationship. In turn, some grandmothers indicated that they would withdraw their support and stop babysitting if their routines and practices met with criticism from the child’s parents. This dynamic could be explained by the socio-cultural norms of respect in Turkish culture, where the older generation is usually highly respected on the basis of their life experience and the wisdom associated with their age [71]. Grandparents in collectivistic cultures, such as the Turkish culture, attach greater importance to conformity (e.g. respect for parents and the elderly, obedience) than to the autonomy which is more highly valued in individualistic cultures, such as the Netherlands [21, 72]. Social expectations of this kind could explain why mothers have a tendency to accept the unsolicited advice and incongruent practices of the grandmothers even though they do not agree with them. Similar findings were found in a Chinese study, which reported that mothers take advice from and behave according to the preferences of older family members, even though they do not agree with them [73]. Besides, except the fact that grandmothers are more authoritative, which encourages dependence and obedience of mothers towards them, mothers need also to rely on grandparents for practical and emotional support in childrearing, which puts them in a powerful position [67].

The results of our study showed that mothers and grandmothers tend to communicate more directly with their own daughter or mother about certain parenting issues and say they have to be far more circumspect in their communication with their daughter-in-law or mother-in-law. Mikucki-Enyart and colleagues also describe the uncertainties sometimes experienced by mothers-in-law regarding their role as grandmother and how they should communicate with their daughters-in-law, about parenting practices for instance [74], and vice versa [75]. Although within collectivistic cultures childcare is seen as a shared responsibility of both kin and non-kin community members, especially grandmothers and experienced women within the community have authority regarding childcare. Grandfathers and fathers does not play an active role regarding childcare during early childhood and rely more on the wisdom, experience and authority of grandmothers on the support they should provide, which point out the significant role of women within these family systems [53].

### Strengths and limitations

The results presented in this paper should be considered in the context of the strengths and limitations of the study. One strength of this study is that it provides insights into the perspectives of both mothers and grandmothers regarding the influence that grandmothers exert on health-related practices during the first 1000 days of a child’s life. This is a relatively unexplored area for this specific target group, and including the perspectives of both mothers and grandmothers enriches our understanding of the issues at stake. Another strength is that two of the researchers are of Turkish origin and resident in the same neighbourhood as the participants. Both were able to communicate fluently with the respondents in Turkish if necessary, which benefited the recruitment and inclusion of mothers and grandmothers. The fact that both researchers shared the same cultural background as the respondents can be seen as both a potential strength and a limitation: on the one hand it might make the respondents feel more comfortable and readily understood during the conversations, but on the other hand, interaction with someone from the same social community might be more likely to elicit socially desirable answers.

A limitation of this study is its use of snowball sampling, which may have limited the scope of the participants to a particular, connected group with a specific set of shared views and values. We tried to limit this effect by also recruiting mothers from various public places. Another limitation is its exclusive focus on female perspectives, due to the decision to only recruit mothers and grandmothers as they are usually the main caregivers. For future studies, we therefore recommend that the perspectives of fathers and grandfathers on their role and influence during the first 1000 days of a child’s life should also be explored. Grandfathers in particular seem to be underrepresented in studies, yet they can also play an influential role in determining health-related practices with regard to young children [76].

## Conclusions and recommendations

This study shows that grandmothers, and indeed the wider social community, play a powerful role in the first 1000 days of a child’s life through their close involvement and engagement with the well-being of young children. While this support is often welcomed by mothers, it can also lead to more detrimental health-related practices with regard to young children and generate tensions between the grandmothers and mothers. Socio-cultural beliefs and practices play a significant role in the contact between parents and the wider community, and we believe that additional research is needed to explore these issues further. It is also important to consider socio-cultural norms in the contact between healthcare professionals and parents, as this can help professionals understand the ideas and beliefs that underlie certain practices. These insights can be beneficial in terms of providing tailored support.

Given the impact that grandmothers and the wider social community have on the lives of parents and young children, we recommend that health-related prevention or education programmes should reflect this topic. Prevention programmes could be developed to specifically target grandmothers and the wider social community of parents, for example by increasing knowledge of certain health issues and by raising awareness of the impact of social pressure on parents and the socio-cultural norms that exert an influence. Health-related prevention programmes that target parents could be expanded to include the issues of negotiating socio-cultural norms and exploring ways to deal with the involvement of other members of the community.

## Supplementary Information

**Additional file 1:** **Appendix A.** Interview guide for grandmothers.

**Additional file 2:** **Appendix B.** Interview guide for mothers.

**Additional file 3:** **Appendix C.** Development of themes.

## Data Availability

The qualitative datasets generated and/or analysed during the current study are not publicly available, as the data contains information that could compromise research participant privacy/consent. However, the data are available from the principal researcher G. Bektas (g.bektas@vu.nl) on reasonable request, and subject to approval from the research committee of Amsterdam UMC (VUmc location).
