# Supplementary material for: A qualitative study on the perspectives of Turkish mothers and grandmothers in the Netherlands regarding the influence of grandmothers on health related practices in the first 1000 days of a child’s life
Source: BMC Public Health. 2022 Jul 16;22:1364. doi: 10.1186/s12889-022-13768-8 (PMC9287533; doi:10.1186/s12889-022-13768-8)
Supplement: Supplementary file 1 — Additional file 1: Appendix A. Interview guide for grandmothers. [file 12889_2022_13768_MOESM1_ESM.docx]

**Appendix A. Interview guide for grandmothers**

- 1. **Pregnancy**
- What advice do you give to pregnant women?
- What traditions or practices are there concerning pregnancy in your culture?
- Which of these traditions still apply today?
- Which traditions have disappeared or changed over time? Why do you think this change has happened?
  1. **Puerperium/Post-partum period**
- What traditional practices are there in your culture during this period? Which of these do you still follow?
- What advice is given to puerperal women in your culture? Which pieces of advice do you give to your daughter (in-law)?
- What traditional practices are there in your culture when it comes to breastfeeding?
- Do you encourage your daughter (in-law) to breastfeed? How do you do that?
- Do you discuss breastfeeding with your daughter (in-law)?
- Is there any difference in how things are now and how they were in the past? If so, what are they?
  1. **Role of grandparents**
- To what extent are you involved in the care of your grandchild?
- What is the role of your husband (i.e. grandfather) in childcare/child rearing?
- How were children looked after in the past? What role did the grandparents play in taking care of their grandchildren?
- What differences do you see in the role/involvement of grandparents in child rearing compared to the past?
- How do you feel about fulfilling this role?
- Which aspects do you feel responsible for (teaching them norms/values, making sure they grow up healthy, eat healthily, sleep enough, play outside etc.)?
  1. **Caring for a baby (including feeding, sleeping etc.)**
- In your culture, what traditional foods/dishes were given to babies when they start eating solid food?
- What is the situation today? Are babies fed differently now than they were in the past? If so, what differences do you see?
- What agreements have you made with your grandchild’s parents about the child’s diet?
- Do you discuss what you can give your grandchild to eat with the parents? What rituals or practices do you have when it comes to your grandchild’s sleep?
- How do you let your grandchild sleep? Do you stick to a fixed sleep schedule set by the mother or do you keep a sleep schedule of your own?
- Are there any specific customs in your culture when it comes to sleeping?
- What kind of activities do you do with your grandchild? Do you play together? If so, how do you play? Do you go outside with the baby?
- How do you deal with screen use (e.g. TV, tablet, phone)? Do you let your grandchild use a screen? If so, when, for what kind of activity and how much screen time is your grandchild/are your grandchildren allowed?
  1. **Ideas about what constitutes a healthy baby**
- How would you describe a healthy baby?
- Does this description reflect the norm in Turkish culture?
- Has the idea or image of a healthy baby changed over time?
- What do your children think about these things? Does their idea or image of a healthy baby differ from yours?
  1. **Communication between grandmothers and mothers**
- Do you have disagreements with your daughter/son (in-law) about childcare?
- About which topics do you have the most discussion?
- What do you do when you find yourself disagreeing? How do you resolve these situations?
