# Supplementary material for: A qualitative study on the perspectives of Turkish mothers and grandmothers in the Netherlands regarding the influence of grandmothers on health related practices in the first 1000 days of a child’s life
Source: BMC Public Health. 2022 Jul 16;22:1364. doi: 10.1186/s12889-022-13768-8 (PMC9287533; doi:10.1186/s12889-022-13768-8)
Supplement: Supplementary file 2 — Additional file 2: Appendix B. Interview guide for mothers. [file 12889_2022_13768_MOESM2_ESM.docx]

**Appendix B. Interview guide for mothers**

**Pregnancy**

- Was it important for you to follow certain Turkish cultural traditions during your pregnancy?
- Were there any family traditions that are often followed during pregnancies? Who introduced these traditions?
- Were your parents (in-law) involved during your pregnancy? Could you explain how they were involved? How did you feel about their involvement?
- Did you ask your parents (in-law) for advice or receive any advice from them during this period?
- Did your parents (in-law) advise you to do certain things during your pregnancy? What did you think of this advice?

**Puerperium/post-partum period**

- Which (traditional) practices often take place during the postnatal period in Turkish culture?
- Did you engage in certain practices or follow certain pieces of advice after you gave birth? Could you explain what they were?
- What do you think about these practices and traditions?
- How important is engaging in these practices and traditions for you? And how important is it to your parents (in-law)? How did they express this?
- Did your parents (in-law) have any opinions about breastfeeding? Did they influence any decisions you took about breastfeeding? Did you get any comments about any of the decisions you made?
- Were certain aspects of breastfeeding particularly important to them?

**Role of grandparents**

- How do you perceive the role of grandparents with regard to child rearing and their influence on health-related behaviours in their grandchildren?
- Do you see grandparents as an extension of the parental role or do you have a different take on this?
- What advice on child rearing given by your parents (in law) did you implement and can you explain why?
- Are there things you do differently from your own parents? Can you explain what they are and why you do them differently?

**Care for the baby (including feeding, sleeping etc.)**

- When your parents (in-law) are babysitting your child, are there rules you think it’s important for them to follow with regard to feeding practices, sleep, physical activity and screen time?
- Do you make arrangements with them about these things? If so, how do you tell them?
- Do you provide them with some sort of schedule (e.g. for feeding or sleeping)?
- When you make arrangements regarding health-related behaviours, do your parents (in-law) keep to them? When do they/don’t they comply with your wishes and why do you think they decide to do so/not do so?
- How do your parents (in-law) respond to the ‘rules’ you set? Do they follow them? Why not? Are you able to discuss your wishes with them?

**Ideas about what constitutes a healthy baby**

- How would you describe a healthy child?
- Does this description reflect the norm in Turkish culture?
- Has the idea or image of a healthy baby changed over time?
- Do you ever get remarks from your parents (in-law) about your child’s weight or appearance? How do you deal with these remarks?

**Communication between grandmothers and mothers**

- Do you ask your parents (in-law) for advice on caring for your child? On which aspects of childcare do you usually ask for advice?
- Do you have disagreements with your parents (in-law) regarding childcare?
- Which topics generate the most discussion?
- What do you do in cases where you disagree? How do you resolve these situations?
