# Supplementary material for: A qualitative study on the perspectives of Turkish mothers and grandmothers in the Netherlands regarding the influence of grandmothers on health related practices in the first 1000 days of a child’s life
Source: BMC Public Health. 2022 Jul 16;22:1364. doi: 10.1186/s12889-022-13768-8 (PMC9287533; doi:10.1186/s12889-022-13768-8)
Supplement: Supplementary file 3 — Additional file 3: Appendix C. Development of themes. [file 12889_2022_13768_MOESM3_ESM.docx]

**Appendix C. Development of themes**

| Final themes | Initial themes | Categories |
| --- | --- | --- |
| 1.The support and cogent advice of grandmothers and the wider social community during the first 1000 days is self-evident and often rooted in socio-cultural beliefs and practices | Rearing child is community task | - rearing child for the community - unsolicited well-meant advices - right to speak - meet the demands of their social environment |
|  | Grandmothers’ involvement | - help and support from grandparents - intensive involvement in 40 days post-partum - indulge grandchild with food or television/tablet/ smartphone - right to spoil |
|  | Ideas/perceptions about healthy child in Turkish culture | - well-fed - perceptions about healthy weight - associate healthy baby with chubby baby - preference for chubby - baby should not be hungry |
|  | Normative assumptions of the sociocultural environment | - critics about weight of child - thinness is associated with - illness - social pressure to have chubbier babies - thin baby associated with not caring well - crying baby is not well-fed/hungry - letting baby cry is unacceptable - expectations to respect elderly people |
| 2. Grandmothers and the wider social community actively encourage mothers to breastfeed | Importance of breastfeeding | - breastfeeding is right of a child - best nutrition for baby - religious funding - wealth of motherhood |
|  | Worries about saturation of breastfed child | - uncertain about saturation - afraid for malnutrition - baby should be well-fed - extra bottle feedings - amount and quality of breastfeeding - crying means hungry |
|  | Strategies to increase breastmilk | - critics on mothers that stops with breastfeeding - providing sweet food to increase milk production - stimulate mothers to breastfed as much as possible |
| 3. Grandmothers often deviate from their grandchildren’s daily routine when they are babysitting | Differences ideas between grandmothers and mothers | - mothers are more Dutch-oriented - own ideas about rearing up a healthy child - trusting own experience and knowledge - routines from past does not harm - routines from past are better |
|  | Grandmothers follow their own routine | - disagreement with practices of parents - following own routine - doing what they are used - feeling guilty/sorry when they do not indulge in grandchild’s wishes - providing food that is not allowed by parents |
| 4. The communication between mothers and grandmothers about differences of opinion regarding health-related practices is perceived as complicated | Grandmothers’ expectations | - expectations to respect their advice and knowledge - being grateful for their help - respect for parents (in-law) |
|  | Mothers‘ dependency | - role of relationships - familial relations - distribution of power - distribution of responsibilities - dependency of parents |
